# Chemical Characterisation of Silanised Zirconia Nanoparticles and Their Effects on the Properties of PMMA-Zirconia Nanocomposites

**DOI:** 10.3390/ma14123212

**Published:** 2021-06-10

**Authors:** Saleh Zidan, Nikolaos Silikas, Suhad Al-Nasrawi, Julfikar Haider, Abdulrahman Alshabib, Alshame Alshame, Julian Yates

**Affiliations:** 1 Department of Dental Materials, Faculty of Dentistry, Sebha University, Sebha 18758, Libya; 2 Division of Dentistry, School of Medical Sciences, Faculty of Biology, Medicine and Health, University of Manchester, Manchester M13 9PL, UK; nikolaos.silikas@manchester.ac.uk (N.S.); j.haider@mmu.ac.uk (J.H.); julian.yates@manchester.ac.uk (J.Y.); 3 Department of Restorative Dentistry, Faculty of Dentistry, University of Kufa, Najaf 54001, Iraq; suhad.alnasrawi@uokufa.edu.iq; 4 Department of Engineering, Manchester Metropolitan University, Manchester M1 5GD, UK; 5 Department of Restorative Dentistry, College of Dentistry, King Saud University, Riyadh 11362, Saudi Arabia; Abdalshabib@ksu.edu.sa; 6 Department of Oral Surgery, Faculty of Dentistry, Sebha University, Sebha 18758, Libya; Als.alshame@sebhau.edu.ly

**Keywords:** dentistry, prosthodontics, PMMA, zirconia (ZrO_2_), coupling agent, silane, nanocomposite, denture base, flexural strength, flexural modulus, hardness

## Abstract

*Objectives*: The objective of this study was to investigate the mechanical properties of high-impact (HI) heat-cured acrylic resin (PMMA) reinforced with silane-treated zirconia nanoparticles. *Methods*: Forty-five PMMA specimens reinforced with zirconia were fabricated and divided into three groups: Pure HI PMMA (control group), PMMA reinforced with 3 wt.% of non-silanised zirconia nanoparticles and PMMA reinforced with 3 wt.% of silanised zirconia nanoparticles. Silanised and non-silanised zirconia nanoparticles were analysed with Fourier Transform Infrared (FTIR) Spectroscopy. For measuring the flexural modulus and strength, a Zwick universal tester was used, and for surface hardness, a Vickers hardness tester were used. Furthermore, raw materials and fractured surfaces were analysed using Scanning Electron Microscopy (SEM). A one-way ANOVA test followed by a post-hoc Bonferroni test was employed to analyse the data. *Results*: The results showed that the mean values for flexural strength (83.5 ± 6.2 MPa) and surface hardness (20.1 ± 2.3 kg/mm^2^) of the group containing 3 wt.% treated zirconia increased significantly (*p* < 0.05) in comparison to the specimens in the group containing non-treated zirconia (59.9 ± 7.1 MPa; 15.0 ± 0.2 kg/mm^2^) and the control group (72.4 ± 8.6 MPa; 17.1 ± 0.9 kg/mm^2^). However, the group with silanised zirconia showed an increase in flexural modulus (2313 ± 161 MPa) but was not significantly different (*p* > 0.05) from the non-silanised group (2207 ± 252 MPa) and the control group (1971 ± 235 MPa). *Conclusion*: Silane-treated zirconia nano-filler improves the surface hardness and flexural strength of HI PMMA-zirconia nanocomposites, giving a potentially longer service life of the denture base.

## 1. Introduction

Acrylic resins remain the preferential choice for complete dentures as they possess satisfactory properties [1] and provide a satisfactory outcome for patient [2]. The most common prosthetic acrylic resin is polymethyl methacrylate (PMMA) [3] or a modified version with a butadiene styrene rubber compound to form high-impact denture bases with improved strength [4,5]. This modification has proved successful for improving impact strength [6]; however, other studies have reported a decrease in the flexural strength [2] and stiffness [6]. Still, in clinical practice, denture bases have demonstrated a tendency to flex during mastication, subjecting the polymer to forces that can cause crack propagation and, eventually, failure of the denture [7].

The incorporation of inorganic nanoparticle fillers into HI PMMA acrylic resin has been suggested as a potential solution to this problem. The incorporation of nanoparticles into polymers has created a novel set of materials that perform better than polymers filled with micro-particles [8]. The effect of these nanoparticles on the mechanical properties of the material varies with particle size, polymer–particle interfaces, methods of manufacture and homogeneity of particle dispersion within the polymer matrix [9,10]. Nanomaterials have a high specific surface area and perform excellently, offering properties that are often radically different from conventional materials [9].

Attempts have been made to enhance the mechanical properties of acrylic resins by incorporating different metal oxide nanoparticles including titanium oxide [8,11], alumina [12,13,14,15] and silica [1]. Zirconium oxide (zirconia, ZrO_2_) is a metal oxide that has attracted interest as an inorganic filler material for developing polymer nanocomposites [16]. The incorporation of zirconia nanoparticles has been investigated and found to enhance some mechanical properties of PMMA resins [12,15,16,17,18]. Zirconia has beneficial mechanical properties such as high flexural strength of 900 to 1200 MPa, a hardness of 1200 HV and a fracture toughness (9–10 MPa.m^1/2^). It is also resistant to corrosion and is biocompatible, making it an excellent option for polymer reinforcement in denture base applications [19].

As nanoparticles exhibit high surface energy, high polarity and hydrophilic surfaces, uniform distribution throughout the polymer matrix can be challenging, causing some phase separation and aggregation [20,21,22]. The surface of the nanoparticles must be modified with coupling agents such as silane [21], which can create a strong interfacial bond with the resin matrix [18,23]. A silane coupling agent is required to retain fillers in the resin matrix. Silane possesses the ability to form chemical bonds with different substrates (inorganic fillers and organic resin matrix) due to its two (or more) functional groups [24]. There are two ways of modifying nanoparticles. One method involves chemical treatment whereby small molecules of the coupling agent are absorbed on the nanoparticle surface [20,25]. The second method of modification can be performed by a graft of polymeric molecules, such as poly-methyl methacrylate, to the hydroxyl groups present on the surface of the nanoparticles through covalent bonds leading to an improved adhesion [20,26].

According to previous research, which evaluated conventional PMMA acrylic resin reinforced with metal oxides, silanisation improved the properties [11,18]. Therefore, it could be useful to investigate the advantages of silanisation in this study. To the authors’ knowledge, the literature has no data available on the effect of zirconia nanoparticle silanisation on the mechanical properties of zirconia impregnated HI PMMA nanocomposites. The purpose of this study was to investigate how silanisation of zirconia nanoparticles affect the flexural strength, flexural modulus and surface hardness of HI PMMA reinforced with 3 wt.% zirconia.

The hypothesis was that there would be no significant difference between the flexural strength, flexural modulus and surface hardness of silanised and non-silanised PMMA-zirconia nanocomposites.

## 2. Materials and Methods

### 2.1. Materials

High-impact, heat-cured, acrylic resin polymethyl methacrylate (PMMA) powder and methyl methacrylate liquid monomer (MMA; product no. 202-327-6) (HI Metrocryl, Metrodent Limited, Huddersfield, UK) were used for the denture base material. Yttria-stabilised zirconia (ZrO_2_) nanoparticles (Product no. 8522QI, Sky Spring Nano Materials, Houston, TX, USA) were used as the filler material for manufacturing the experimental specimens.

### 2.2. Specimen Preparation

Zirconia nanoparticle surfaces were treated with 7 wt.% silane coupling agent (3-trimethoxysilyl propyl methacrylate; product no. 440159, Sigma Aldrich, Gillingham, UK) as explained in the Author’s previous publication [27].

According to previous studies, a small percentage addition (1% to 7.5 wt.%) of different metal oxide nanoparticles can enhance the mechanical properties of PMMA denture base resin [11,14,15,18]. Therefore, a low concentration of zirconia nanoparticles (3 wt.%) was used to prepare nanocomposite specimens for this study.

An electronic balance (Ohaus Analytical plus, Ohaus Corporation, Parsippany, NJ, USA) was used to weigh the silane-treated and non-silane zirconia and acrylic resin powders according to Table 1. The zirconia was added to the acrylic resin monomer and hand-mixed using a spatula to ensure uniform distribution of powder throughout the monomer and to avoid any aggregation. The HI acrylic resin powder was then mixed with the liquid until uniform, as per the manufacturer’s instructions (Metrodent). Upon reaching the dough-like stage that allowed handling, it was pressed into a mould by hand. The closed mould was then inserted in a hydraulic press (Sirio P400/13045) with a pressure of 15 MPa and with any excess mixture being removed. The mould was then submerged in a curing bath for 6-hour polymerisation at a temperature of 95 °C. The mould was then removed from the curing bath and left to cool for 30 min at room temperature. After removal from the mould, specimens were then trimmed using a tungsten carbide bur, ground with an emery paper and polished with pumice powder in a polishing machine (Tavom, Wigan, UK).

**Figure 1:**
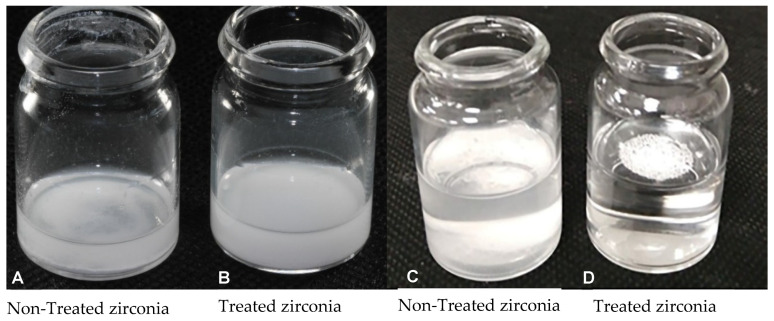
Photographs illustrating non-treated and silane-treated zirconia nanoparticles mixed in (**A**,**B**) monomer (MMA) and (**C**,**D**) water.

In order to understand their behaviours, a comparison between silanised and non-silanised zirconia nanoparticles was assessed in methyl methacrylate monomer (MMA) and in water. In order to ensure an equal number of particles for this test in both liquids, an equal weight of nanoparticles was silanised. In both liquids (MMA and water), silanised particles were homogeneously distributed and remained suspended, and this was in contrast to the non-silanised particles, which appeared to settle at the bottom of the containers (Figure 1). A similar result was also observed by other researchers [28]. Therefore, it is expected that silane functionalisation would encourage the homogeneous distribution of zirconia throughout the nanocomposites.

### 2.3. Materials Characterization

An Energy Dispersive X-ray Spectroscopy (EDX) was used to characterise the elemental compositions of PMMA and zirconia particles. The particles were randomly selected and then loaded into the SEM/EDX (Carl Zeiss Ltd, 40 VP, Smart SEM, Cambridge, UK) for imaging using the secondary electron detector at an acceleration voltage of 20.0 kV. Fourier Transform Infrared spectroscopic (FTIR) (Perkin-Elmer Spectrum Two, Spotlight 200i, Waltham, MA, USA) were used to find the functional groups and bonds in the silanised zirconia nanoparticles powders and non-silanised zirconia. Spectra were obtained with the wavenumber range from 4000 to 400 cm^−1^ at a resolution of 4 cm^−1^ at room temperature [17].

### 2.4. Mechanical Tests

The flexural strength of the specimens was determined using a 3-point bending test in a universal testing machine (Zwick/Roell Z020 Leominster) in accordance with the British International Standard for Denture Base Polymers (2487:1989) [29]. Ten specimens were used for each group with the dimensions 65 ± 1.0 mm length × 10 ± 0.1 mm width × 2.50 ± 0.1 mm thickness. Flexural strength was calculated in MPa for all specimens using Equation (1) [27,29].

(1) $$\sigma =\frac{3\mathrm{Fl}}{2{\mathrm{bh}}^{2}}$$

where F is the maximum force applied in N, l is the distance between the supports in mm, b is the width of the specimen in mm and h is the height of the specimen in mm. The flexural modulus was determined as the slope of the linear portion of the stress/strain curve for each test run.

The Vickers hardness (HV_0.05_) of the specimens was evaluated using a micro-hardness testing machine (FM-700, Future Tech Corp, Tokyo, Japan). Fifteen specimens were divided into five specimens for each group and the dimensions of the specimens were 65 ± 1.0 mm length × 10 ± 0.1 mm width × 2.50 ± 0.1 mm thickness, and the test load was fixed at 50 g for 30 s. During measurement, three indents were taken at different points on one side of each specimen. The distances between the indentations were determined by multiplying the average indentation diagonal length by four (4×D) to ensure adequate distance was maintained between the indentations. The Vickers hardness was calculated by measuring the diagonals (D1, D2) of the pyramid-shaped indentation impressed on the specimen under the applied load. The hardness values were averaged from five specimens for a particular group. Further details of the test procedures are described in [27].

### 2.5. Imaging of Particles and Fractured Surface

The distribution of the particulate size and structure of the HI PMMA powder and zirconia nanoparticles in raw form were characterised by scanning electron microscopy (SEM, Carl Zeiss Ltd, 40 VP, Smart SEM, Cambridge, UK). The surfaces of fractured of the nanocomposites from the 3-point bending test were also studied using SEM. The particles from the raw materials and the fractured nanocomposite specimens were mounted onto aluminium stubs and sputter-coated with a thin layer of gold. The specimens were then loaded into the SEM for imaging using the secondary electron detector at an acceleration voltage of 2.0 kV.

### 2.6. Statistical Analysis of Test Results

The mechanical tests results were recorded and analysed using a SPSS (IBM SPSS statistics version 23, IBM, New York, NY, USA). Descriptive statistics were conducted for each of the tests to determine if the data were normally distributed. According to tests on normality with Kolmogorov–Smirnov and Shapiro–Wilk, the data were normally distributed for all tests. The data from the flexural strength, flexural modulus and hardness tests were analysed by using a one-way analysis of variance (ANOVA) with post-hoc Tukey significant difference test (*p* < 0.05).

## 3. Results

### 3.1. Surface Morphology Analysis of Particles

SEM analysis showed that the PMMA powder had a particle size averaging 50 µm, with a range between 10 µm and 100 µm. Further visible in the powder were the butadiene styrene rubber particles, averaging 30–50 µm. The yttria-stabilized zirconia nanoparticles averaged between 30 nm and 60 nm for individual particles and 200–300 nm when clustered [27].

### 3.2. Chemical Analysis of Particles

**Figure 2:**
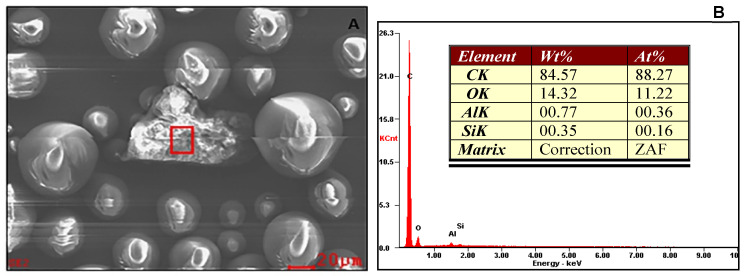

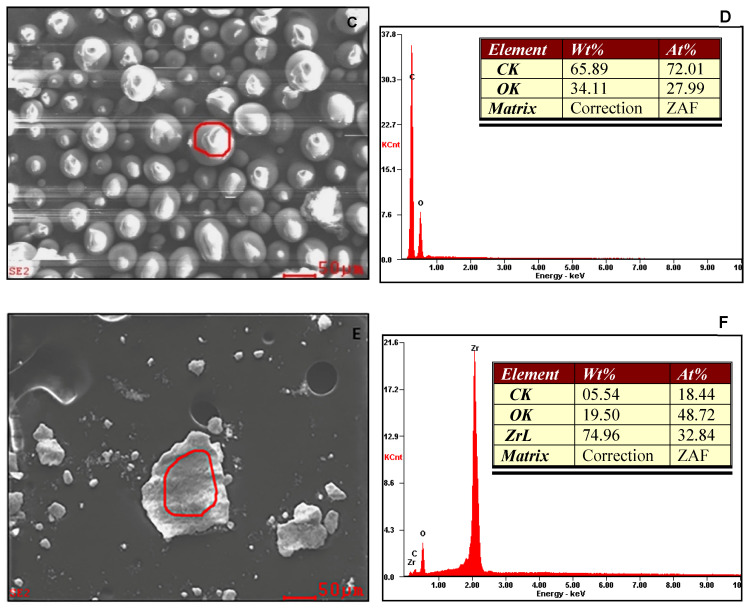
SEM images and EDX spectra representing (**A**,**B**) rubber particles in PMMA powder, (**C**,**D**) PMMA particles and (**E,F**) zirconia nanoparticles.

Figure 2 presents SEM images and EDX spectra of butadiene styrene rubber within PMMA, PMMA particles and zirconia particles. The elemental compositions obtained are also presented in terms of weight and atomic percentages. EDX confirmed rubber particles represented by high percentages of carbon and oxygen with traces of other elements such as Al and Si in contrast to only carbon and oxygen in the PMMA particles. Other than zirconium and oxygen, a noticeable amount of carbon was present in the zirconia nanoparticles.

### 3.3. Analysis of Zirconia Functionalising by Silane 

**Figure 3:**
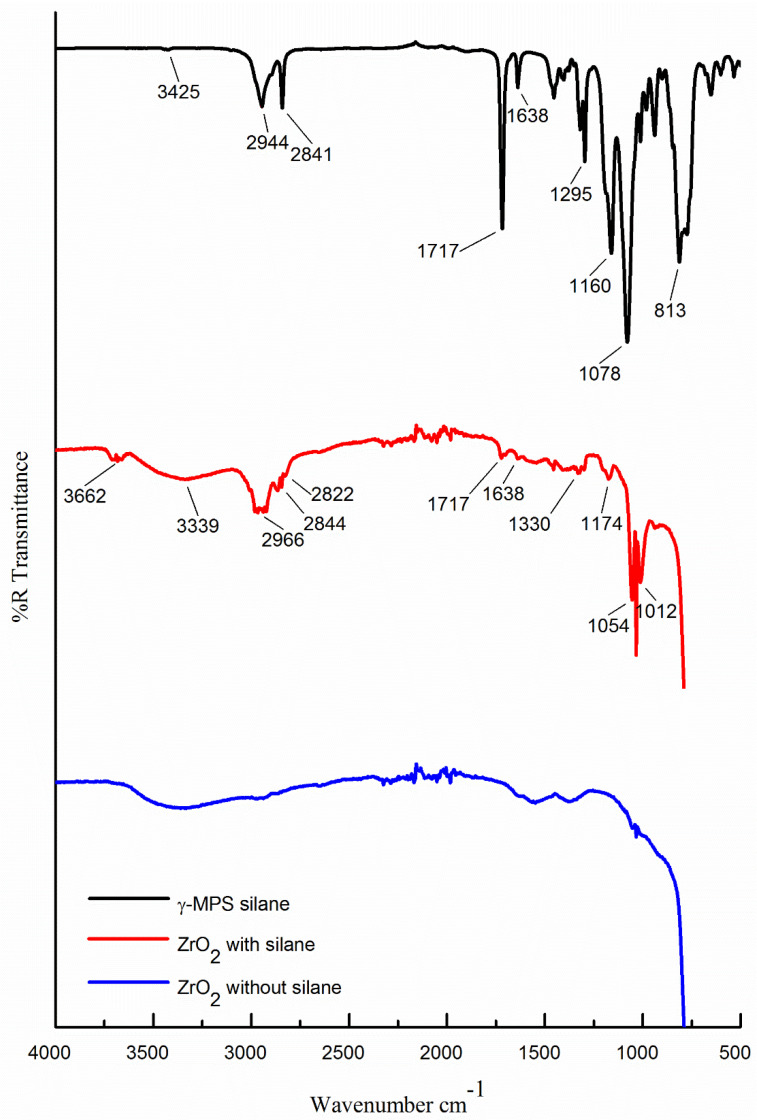
FTIR spectra showing (black curve, top) silane coupling agent (γ-MPS), (red curve, middle) zirconia nanoparticles after treatment with silane (γ-MPS) and (blue curve, bottom) zirconia nanoparticles without silane.

Figure 3 illustrates the FTIR spectrum structure of silane coupling agents (γ-MPS) and presents the spectra curves of pure zirconia (without silane) and silanised zirconia. Both the zirconia spectra curves displayed the absorption peak between 3650 and 3200 cm^−1^ associated with stretching vibrations of OH (hydroxy group) on the surface of the zirconia nanoparticles, [13,17], with a broader absorption peak in silanised zirconia.

In the spectral curve of γ-MPS, the peaks between 2944 and 2841cm^−1^ exhibited the asymmetric and symmetric vibrations of CH_3_ and CH_2_ groups. In contrast, the curve relating to silanised zirconia demonstrated absorption peaks around 2966 and 2844 cm^−1^. The absorption peak in the γ-MPS curve at approximately 1717 cm^−1^ was attached to the C=O group (carbonyl) vibrations of the (γ-MPS) molecules [8]. An absorption peak observed on the surface of silanised zirconia at 1717 cm^−1^ indicated the formation of C=O group vibrations. The absorption peak at 1638 cm^−1^ was attributed to the C=C group in the γ-MPS on the silanised zirconia surface. The characteristic peaks between 1160 and 1078 cm^−1^ in the γ-MPS spectrum represented vibration absorption bands of Si-O-C and Si-O-Si, respectively. A strong peak at 813 cm^−1^ was denoted by the vibration of Si-C [8]. In silanised zirconia, the peaks at 1174 and 1054 cm^−1^ matched Zr-O, while the peaks at 1330 and 1054 cm^−1^ indicated Zr-O-Si. Silane hydrolysis produced silanol groups that condensed with surface hydroxyl groups on the inorganic particles (ZrO_2_) to form covalent bonds [30]. The FTIR analysis confirmed successful grafting of the coating of silane (γ-MPS) onto the surface of zirconia nanoparticles. Table 2 lists all bond types and wavelengths.

**Figure 4:**
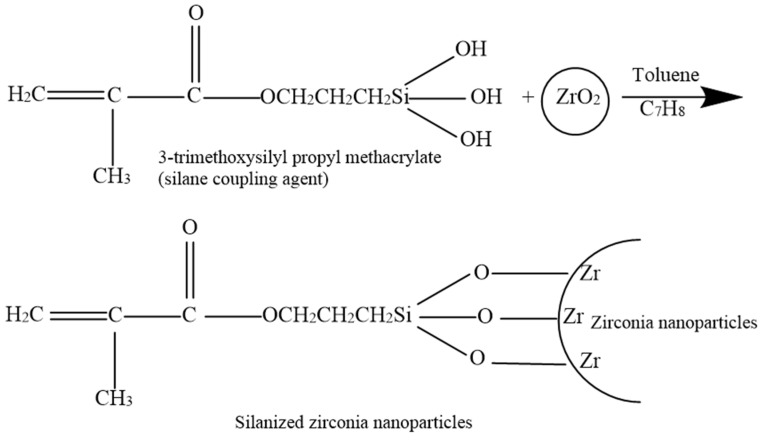
Illustration showing the chemical bonding of silane (γ-MPS) with zirconia (ZrO_2_) nanoparticles.

The mechanism by which the 3-trimethoxysilyl propyl methacrylate silane coupling agent reacts with the zirconia nanoparticles’ surface and the bonding with PMMA is presented in Figure 4 [28,31].

### 3.4. Flexural Strength and Modulus

**Figure 5:**
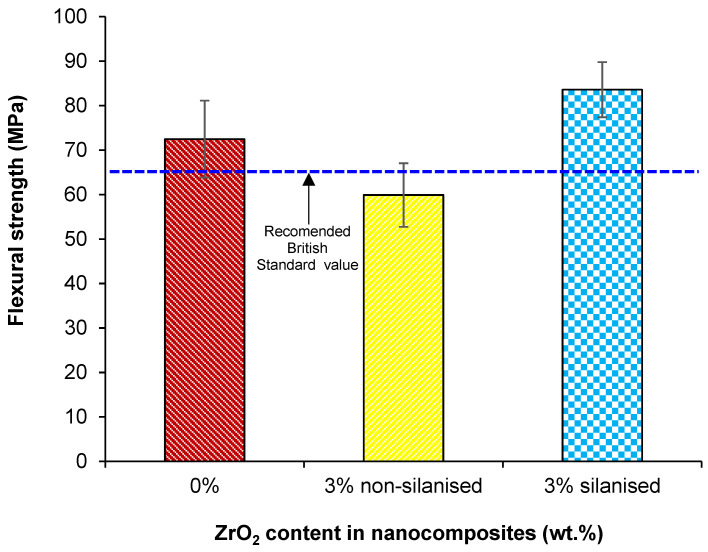
Bar charts showing the effect of treating zirconia nanoparticles on the flexural strength of PMMA-zirconia nanocomposites.

**Figure 6:**
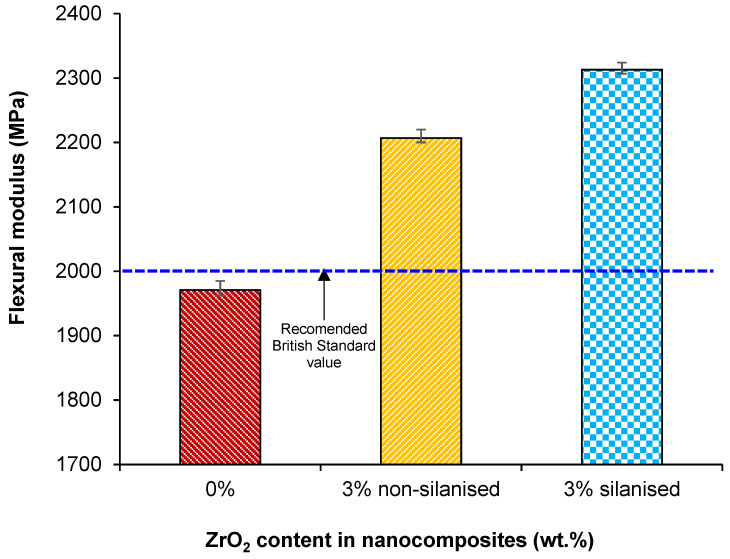
Bar chart showing the effect of treating zirconia nanoparticles on the flexural modulus of the PMMA-zirconia nanocomposites.

The mean and standard deviation values of flexural strength and modulus are given in Table 3 and Figure 5 and Figure 6. One-way ANOVA indicated a statistically significant difference between the mean values (*p* < 0.001) with the Tukey test showing a significant difference (*p* < 0.05) between test groups of silanised and non-silanised nanocomposites and the control group. However, flexural modulus values showed no significant difference (*p* > 0.05) between silanised and non-silanised specimens. 

The highest mean value of flexural strength was (83.5 ± 6.2 MPa) for the silanised group compared to the non-silanised group (59.9 ± 7.1 MPa). The reinforcement (3 wt.%) of HI PMMA with silanised zirconia nanoparticles increased flexural strength by 40% and 15% compared to the non-silanised group and control group, respectively. The highest mean value of flexural modulus was found (2313.0 ± 161.3 MPa) for the group with 3 wt.% of silanised ZrO_2_ representing a 5% increase in the modulus compared to the non-silanised group (2207.0 ± 252.7 MPa) but was not significantly different (*p* < 0.05). When compared to the control, this represented an increase in the flexural modulus of 17%. 

### 3.5. Vickers Hardness

**Figure 7:**
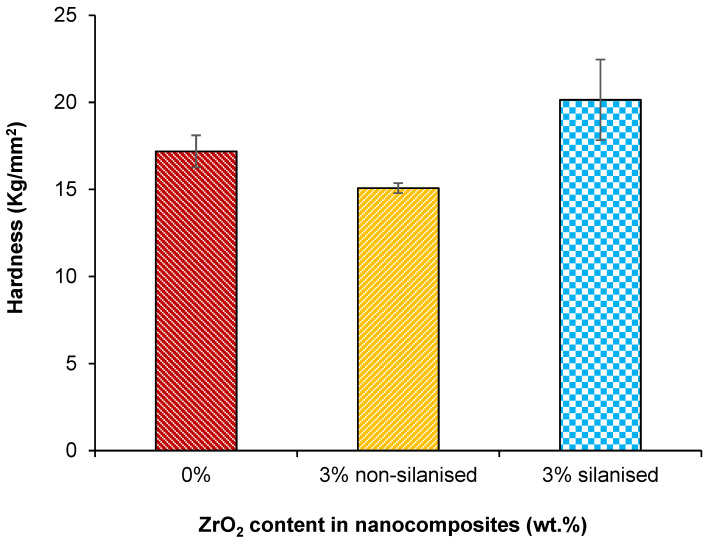
Bar chart showing the effect of treating zirconia nanoparticles on the hardness of the PMMA-zirconia nanocomposites.

The mean and standard deviation surface hardness values for each experimental nanocomposite are listed in Table 3 and Figure 7. The Tukey test indicated a significant difference (*p* < 0.05) between the silanised and non-silanised nanocomposites and control group. The highest value of Vickers hardness was obtained (20.1 ± 2.3 kg/mm^2^) for the group with 3 wt.% silanised zirconia, and was significantly different (*p*< 0.05) in comparison to the non-silanised (15.0 ± 0.2 kg/mm^2^) and control (17.1 ± 0.9 kg/mm^2^) groups, representing a 33% and 17% increase in the surface hardness, respectively.

### 3.6. Fractured Surface Analysis

**Figure 8:**
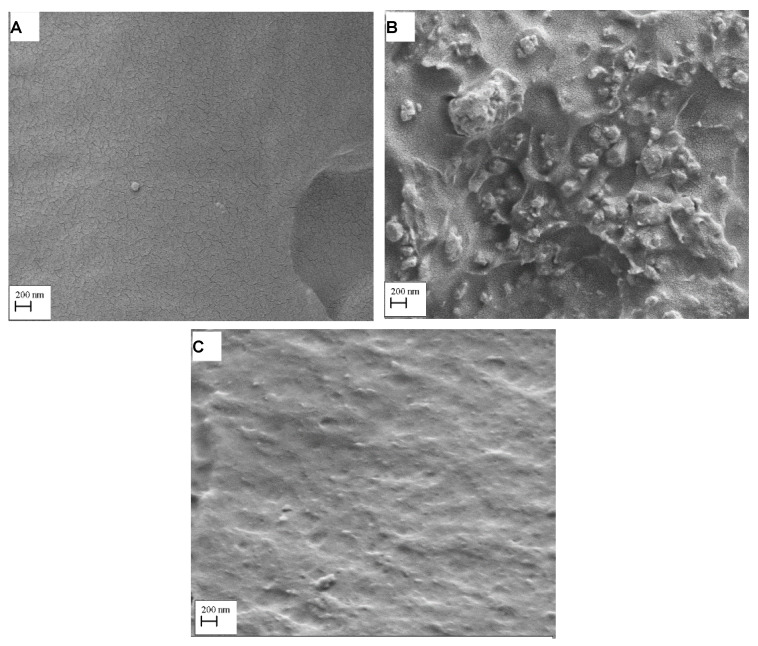
SEM micrographs detailing the fractured surfaces of (**A**) pure HI PMMA (**B**) PMMA-zirconia nanocomposite with non-treated zirconia and (**C**) PMMA-zirconia nanocomposite with silane-treated zirconia.

SEM micrographs in Figure 8 show the fractured surfaces of PMMA-zirconia nanocomposites with silanised and non-silanised zirconia. Zirconia particles were visible in the PMMA matrix, but specimens with silane-treated zirconia showed less cluster formation compared to the specimen with non-treated zirconia. 

## 4. Discussion

Silanisation of zirconia nanoparticles can enhance the mechanical properties of HI PMMA-zirconia nanocomposites. The hypothesis was therefore rejected, as significant differences in the surface hardness and flexural strength of the nanocomposites with silanised zirconia were found compared to both the nanocomposite with non-silanised zirconia and the control group. However, the hypothesis was partially accepted as there was no significant difference in flexural modulus between silanised and non-silanised groups.

According to the British International Standard for Denture Base Polymers (2487: 1989) [29], flexural strength and modulus should not be lower than 65 MPa and 2000 MPa. In the current study, the values for flexural strength of the silanised group (83.5 MPa) and control group (72.4 MPa) were acceptable. However, the flexural strength of the non-silanised group (59.9 MPa) was not acceptable according to the aforementioned British Standard. In the current study, silane treatment of zirconia nanoparticles in HI PMMA composites improved the flexural strength by 40% and 15% when compared to the non-silanised and control groups. The findings agreed with a previous study demonstrating that the inclusion of silanised zirconia nanoparticles in a repaired denture-base resin enhanced flexural strength [18]. It was pointed out that the reason for the increased flexural strength may be related to the nanoparticle sizes, their distribution within the matrix resin and the silanisation method used.

A similar study by Zhang et al. investigated reinforcement of PMMA denture bases with silanised zirconia nanoparticles and aluminium borate at different concentrations and their effects on flexural strength [15]. They found that 2 wt.% and 3 wt.% of silanised zirconia significantly improved the flexural strength of PMMA. Vojdani et al. showed that flexural strength significantly increased in with the addition of 2.5 wt.% aluminium oxide to a conventional PMMA denture-base resin [14]. Similar behaviour was also observed by Elshereksi et al. [8] in composites with PMMA and nanobarium titanate. Da Silva et al. reported significantly higher flexural strength for microwave-heat-cured (PMMA) denture-base resin reinforced with 0.5 wt.% and 1.0 wt.% silanised silica compared to pure PMMA and suggested the underpinning mechanism in the increase of the flexural strength could be due to use of a microwave curing process instead of a water bath [1]. A different study by Ergun et al., who investigated zirconia nanoparticles at different concentrations (5%, 10%, 20 wt.%) impregnated into conventional PMMA acrylic resin, demonstrated that the flexural strength decreased with an increase in zirconia percentages in contrast to pure PMMA. Heterogeneous dispersion of nanoparticle fillers throughout the resin matrix and filler clustering were reported as the reasons for the decrease in strength [9].

The increase in flexural strength noted in the present study might be explained by the fact that the reinforcement of the HI PMMA acrylic resin matrix with silanised zirconia nanoparticles caused better adhesion of the particles and the matrix facilitated by favourable chemical bonding due to silanisation. Silane (γ-MPS) has a bifunctional molecule that can react by its alkoxysilane groups with the filler and itself, and with the resin due to its methacrylate functional group [32]. According to Karabela et al., who explored the effect of silane molecules on dental resin nanocomposites, during silanisation of silica nanoparticles, the -OCH_3_ functional groups in silane molecules were hydrolysed to silanol groups by moisture containing solvent. These silanol groups formed covalent bonds upon condensing with surface hydroxyl groups on the silica [33]. During silanisation, a film of multiple layers of silane molecules was formed around the filler particles, which was either chemically or physically attached to the filler particles [34]. In addition, the carbonyl groups of silane and the hydroxyl groups of silica formed hydrogen bonds [33].

Lung et al. concluded that the rate of silane hydrolysis is dependent on multiple factors such as temperature and type of solvent used for hydrolysis activation [24]. In the current study, during the silanisation procedure, silane (γ-MPS) was dissolved in toluene solvent before its incorporation into the monomer. After the silane hydrolysis and condensation had taken place on the surface of zirconia nanoparticles, an EZ-2 Elite personal solvent evaporator machine was used for drying the samples at 80 °C for 3 hours, which increased the stability of the silane coating on the surface of zirconia, as indicated by the FTIR analysis in Figure 3, compared to the non-treated zirconia.

The flexural modulus values varying between 2207 MPa to 2313 MPa for both silanised and non-silanised nanocomposites was acceptable according to the British Standard. However, the flexural modulus value was 1971 MPa for the control group and was not acceptable. The modulus value is slightly lower than the recommended values and the reason for the lower value could be explained by the fact that PMMA reinforced with rubber particles increases the impact strength but reduces the flexural modulus [6]. Alhareb et al. explored the effect of incorporating 7.5 wt.% nitrile butadiene rubber as an impact modifier together with Al_2_O_3_/YSZ particles at varying concentrations (1, 3, 5, 7, 10 wt.%) into a PMMA denture base. The findings demonstrated that an increase in fillers improved the flexural modulus [12]. In addition, some previous studies assessed the impact of a silane surface treatment of hydroxyapatite particles used to reinforce PMMA resins and found the flexural modulus to increase. The reason for improvement was attributed to silane, which enhanced the interfacial interaction between the PMMA and hydroxyapatite particles [34,35,36]. The results of the presented study match those observed in earlier studies: The flexural modulus of silanised nanocomposites increased by 5% compared to the non-silanised group in contrast to a significant increase (17%) compared to the control.

High hardness values commonly correspond to higher resistance to abrasion [10]. It is interesting to note that the surface hardness of silanised nanocomposites significantly increased with the silane treatment compared to the non-silanised and control groups. Zhang et al. documented that the surface hardness of PMMA with 3 wt.% of zirconia and aluminium significantly improved compared to the nanocomposite group containing 3 wt.% of silanised zirconia in this study [15]. Lung et al. evaluated the effect of adding non-treated and silane-treated hydroxyapatite fillers to dental resin and found that silanisation of hydroxyapatite particles increased surface hardness of the composite [24]. This increase was attributed to the higher rigidity of the filler particles than the resin matrix along with strong chemical bonds formed between the matrix and nanoparticles, which required more energy to break these linkages. This explanation was in agreement with the current study that confirmed that adhesive silane coating was formed on the surface of zirconia nanoparticles as observed in FTIR analysis (Figure 3). Moreover, the surface hardness of conventional PMMA resin increased with the increase of zirconia nanoparticle concentration [1,9,10].

Alhotan et al. evaluated the flexural strength and surface hardness of heat-cured PMMA modified by the addition of ZrO_2_ nanoparticles with different concentrations (wt.%). The finding showed that the 3–5 wt.% of ZrO_2_ nanoparticles increased flexural strength and surface hardness, which was in agreement with the current study [37]. In relation to the microscopic structure, in the present study, SEM image analysis in Figure 8 shows non-homogenous dispersion and cluster formations of nanoparticles on a fractured surface in the non-silanised HI PMMA-zirconia nanocomposites, which could be one of the reasons for the decreased flexural strength, flexural modulus and hardness. The specimen group with silanised zirconia displayed no significant clustering and good distribution of silanised nanoparticles within the HI PMMA matrix. Additionally, improved adhesion between nanoparticles and the resin matrix could result in significantly improved mechanical properties. Consequently, a combination of homogenous particle distribution and adhesion with the matrix can prevent crack propagation through the PMMA. This is known as dispersion strengthening, which is achieved by adding a second phase of ZrO_2_ nanoparticles to the PMMA matrix to achieve higher strength [16].

One of the limitations of the present report could be that the ageing effect on the stability of the chemical bonding between the zirconia nanoparticles and acrylic resin after immersion in water for a long time was not evaluated by measuring the flexural strength or hardness. Some variables could alter the results of the present work. In fact, wear [38] or long-term brushing [39] could alter both mechanical characteristics and surface smoothness of the dental materials. Therefore, further investigations are needed in order to take into careful account of these clinical conditions. Homogeneity of particle dispersion can be quantitatively measured using polydispersity index (PDI) [40].

Silanisation of zirconia nanoparticles is important in the clinical practice for denture base application to enhance the mechanical properties of HI PMMA-zirconia nanocomposites through strong filler bonding with the resin matrix. This study suggested that HI PMMA incorporating 3 wt.% of silanised zirconia nanoparticles could additionally improve the flexural strength and hardness possibly due to the uniform dispersion of the nanoparticles and good bonding with the PMMA matrix in clinical use when compared with the non-silanised zirconia, leading to a longer operating life of the denture.

## 5. Conclusions

Zirconia (ZrO_2_) nanoparticles were treated with a silane (γ-MPS) coupling agent, and PMMA-based nanocomposites were prepared with 3wt.% treated and non-treated nanoparticles. Silanised and non-silanised zirconia nanoparticles were analysed with Fourier Transform Infrared (FTIR) Spectroscopy and mechanical properties of the nanocomposites were evaluated. Within the limitations of this study, the following conclusions can be drawn.

It was confirmed by the FTIR analysis that a coating of silane was successfully grafted onto the surface of zirconia nanoparticles.

Surface hardness and flexural strength of the denture base nanocomposite based on PMMA and silane-treated 3 wt.% zirconia (ZrO_2_) were improved significantly (*p* < 0.05) compared to the nanocomposite reinforced with non-treated zirconia and pure PMMA (control group) possibly due to uniform distribution of the nanoparticles and good bonding between the nanoparticles and PMMA.

The flexural modulus of nanocomposites with treated zirconia was significantly higher than the control. However, no significant difference was found when compared to the nanocomposite with non-treated zirconia.

Therefore, in order to improve the clinical service life of complete denture, PMMA resin should be reinforced with silane-treated zirconia nanoparticles.

## Figures and Tables

**Table 1:** Components of the experimental specimens by weight.

Experimental Group	Zirconia (wt.%)	Zirconia (g)	HI PMMA Powder (g)	HI MMA Monomer (mL)
Control	0.0	0.000	21.000	10.0
3.0	3.0	0.630	20.370	10.0

**Table 2:** Bond type and wavelength of Silane coupling agent, 3-trimethoxysilyl propyl methacrylate (γ-MPS) and silanised zirconia nanoparticles.

Bond Type	Wavelength (cm^−1^)	Reference of γ-MPS
O-H	3650–3200	3425
CH_3_-CH_2_	2966–2844	2944–2841
C=O	1717	1717
C=C	1638	1638
Zr-O	1174–1054	1160–1078
Zr-O-Si	1330–1054	1160–1078

**Table 3:** Mean and standard deviation (SD) flexural strength, flexural modulus and Vickers hardness values for the test groups.

Specimen Group	Flexural Strength (MPa)	Flexural Modulus (MPa)	Hardness (kg/mm^2^)
	Mean & SD	Mean & SD	Mean & SD
Control (0.0 wt.%)	72.4 (8.6) ^A^	1971.0 (235.0) ^A^	17.1 (0.9) ^A^
Non-silanised (3.0 wt.%)	59.9 (7.1) ^B^	2207.0 (252.7) ^AB^	15.0 (0.2) ^A^
Silanised (3.0 wt.%)	83.5 (6.2) ^C^	2313.0 (161.3) ^B^	20.1 (2.3) ^B^

Note: Within a column, cells having similar (upper case) letters are not significantly different.

## Data Availability

The data presented in this study are available within the article.
